# The top 100 most cited articles on bronchoscopy: a bibliometric analysis

**DOI:** 10.1186/s12890-020-01266-9

**Published:** 2020-08-27

**Authors:** Boxue He, Pengfei Zhang, Qidong Cai, Shuai Shi, Hui Xie, Yuqian Zhang, Xiong Peng, Zhenyu Zhao, Wei Yin, Xiang Wang

**Affiliations:** 1 Department of Thoracic Surgery, The Second Xiangya Hospital, Central South University, Changsha, 410011 Hunan China; 2 Hunan Key Laboratory of Early Diagnosis and Precision Therapy, Department of Thoracic Surgery, The Second Xiangya Hospital, Central South University, Changsha, 410011 Hunan China

**Keywords:** Citations, Most-cited papers, Bronchoscopy

## Abstract

**Background:**

Bronchoscopy is applied broadly in the diagnosis and treatment of pulmonary diseases. Over the past few decades, an increasing number of studies about bronchoscopy have been published. However, little is known about their qualities and characteristics.

**Methods:**

All of the databases in Web of Science (including the Web of Science Core Collection, BIOSIS Citation Index, KCI-Korean Journal Database, MEDLINE, Russian Science Citation Index, and SciELO Citation Index) were utilized to identify articles published from 1990 to 2020. The top 100 most cited articles about bronchoscopy were selected for degree centrality analysis and analyses regarding publication time, total citation number, the citation density, time-related flux, first author, published journal, geographic origin, and research theme.

**Results:**

The selected articles were published mainly in the 2000s and 1990s. Citations per article ranged from 731 to 196. The leading country was the USA, followed by the United Kingdom. The most frequently studied themes were bronchoalveolar lavage (BAL) fluid and biopsy. The degree centrality analysis connoted that “BAL, inflammation, diagnosis” had a high degree of centrality in the 1990s, while “diagnosis, BAL, biopsy, prospective” took centre stage in the 2000s.

**Conclusions:**

The time, area, and theme distribution of the 100 most cited articles on bronchoscopy have been thoroughly analyzed. It is noticeable that researches based on BAL and endobronchial or transbronchial biopsies currently plays a major role.

## Introduction

German laryngologist Gustav Killian performed the first bronchoscopy by using rigid bronchoscopy to remove a pork bone from a patient’s airway in 1897 [1]. The procedure was performed while the patient was awake and cocaine was utilized as a local anesthetic. Nowadays, endoscopy techniques have developed into an approach which can effectively detect and localize critical early pathological changes occurring in the bronchial epithelium and subepithelial regions of human bodies [2]. Bronchoscopies including autofluorescence bronchoscopy, optical fluorescence and reflectance spectroscopy, high-magnification bronchoscopy, high-frequency endobronchial ultrasound, optical coherence tomography, etc. remain a cornerstone in helping identify the etiology of radiographic abnormalities in human bronchial and lung [3]. Under the assistance of bronchoscopies, diseases which can be diagnosed cover chronic lung diseases such as asthma, chronic obstructive pulmonary disease, interstitial lung disease, some lung cancer, and even pulmonary infectious diseases [4–6]. It has aroused our interest that what are the common laws of the complex literature surrounding bronchoscopy these years, and how will it guide future research. Therefore, we hope to carry out a literature-based analysis of bronchoscopy.

There is a unique tool called bibliometrics for analyzing the quality and characteristics of published articles. It was first published in JAMA in 1987 and has been widely used in various fields to assess the importance of published articles or research trends [7]. Through a literature search, we found that there were quite a few highly cited literature (citation time > 150) on bronchoscopy or utilizing bronchoscopy as the main research method, but no literature on bibliometrics analysis yet. The purpose of this study was to investigate the 100 most cited publications in the field of bronchoscopy, to highlight knowledge milestones in the field, and to analyze the quality and characteristics of the most cited original papers of the past 30 years. We also look forward to finding out the most promising research direction about bronchoscopy.

## Methods

### Search strategy and criteria

Web of Science (Thomson Reuters, New York, USA) allows access to more than 12,000 peer-reviewed journals published since 1945, along with their collected citation data [8]. On February 10, 2020, we searched online via all of the databases in Web of Science (including the Web of Science Core Collection, BIOSIS Citation Index, KCI-Korean Journal Database, MEDLINE, Russian Science Citation Index, and SciELO Citation Index). To enhance the sensitivity, two independent researchers used the same search terms (“bronchoscopy” OR “bronchoscopies” OR “bronchoscopic”) with the determined period of 1990 to 2020 and without any language restrictions to search simultaneously. A total of 35,637 results were gathered, and after filtering by Literature type as “articles” and/or “clinical trial”, 29,030 pieces of literature was listed. Thus, only typical original articles were included, and literature such as reviews, meta-analyses, and guidelines was excluded.

**Fig. 1:**
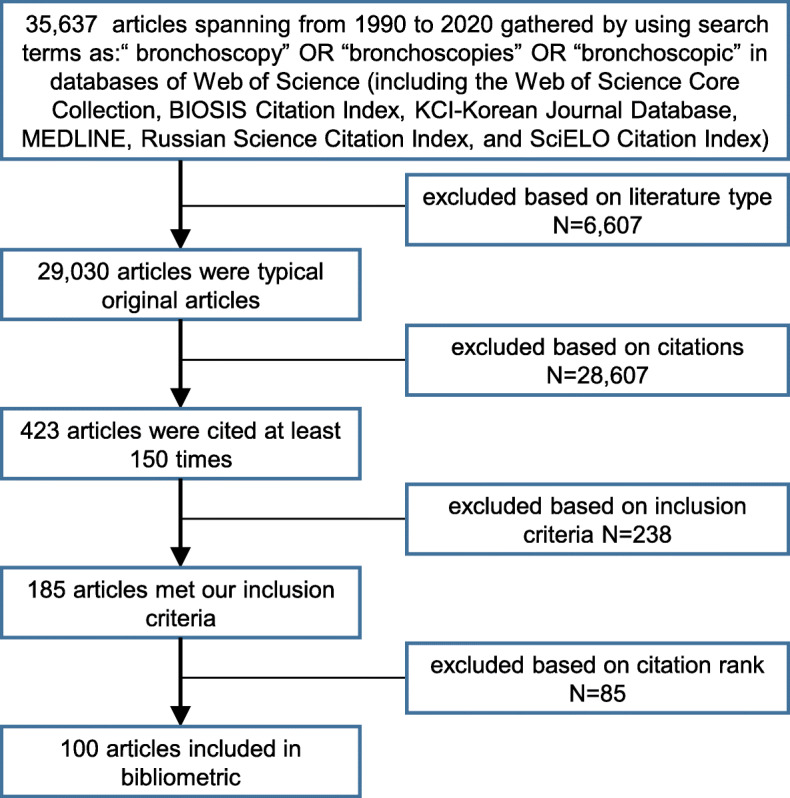
Flowchart illustrating the article allocation process

To reduce the number of articles that need to be screened and improve the gold content of selected documents, we then exported articles that have been cited at least 150 times to EndNote. These 423 articles included were all reviewed by two independent researchers based on the title and the abstract of each. Articles which met the following criteria were included to our study group: (1) The article mainly focuses on bronchoscopy (including rigid bronchoscopy, fiber bronchoscopy, fluorescent bronchoscopy, and so on) with the topic of technical improvement or evaluation; (2) The article compares the advantages or disadvantages of bronchoscopy and other examination or sampling methods; (3) The article mentions bronchial thermoplasty, bronchial valve surgery or other bronchoscope-based operation to solve clinical problems; (4) The article is mainly based on bronchoalveolar lavage (BAL) fluid or bronchoscopy biopsies to get samples for later research. The disagreement between the two researchers was discussed in sequence to reach an agreement. Finally, there remained 185 articles for the following research. These articles were ranked by the number of citations and the top 100 articles were included in this analysis (Fig. 1).

### Extraction

All articles were reviewed by two independent experienced investigators. The following information was listed for all articles: the journal name, year of publication, article age (2020 minus the year of publication), first author, geographic origin, total citation number, the citation density (total citation number/article age), and research theme (introduced later).

### Statistical analysis

The distribution of individual variables for normality was tested using the Shapiro-Wilk test and the Kolmogorov-Smirnov test. Normally distributed data were presented as mean ± standard deviation. A comparison between means was performed using a one-way analysis of variance (ANOVA). Time-dependent trends were tested by the Mann-Kendall trend test. Correlation between variables was performed by the Spearman rank test. *P* < 0.05 was considered to be statistically significant. The analysis was performed using IBM SPSS Statistics, Version 24.0. The Ucinet for windows, version 6.212 was used for the degree of centrality analysis. At last, we used the same search strategy to search 1000 most cited articles in the core data set of Web of Science, and then used HistCite Pro 2.1 software to re-analyze and compare with the former-mentioned 100 articles.

## Results

**Fig. 2:**
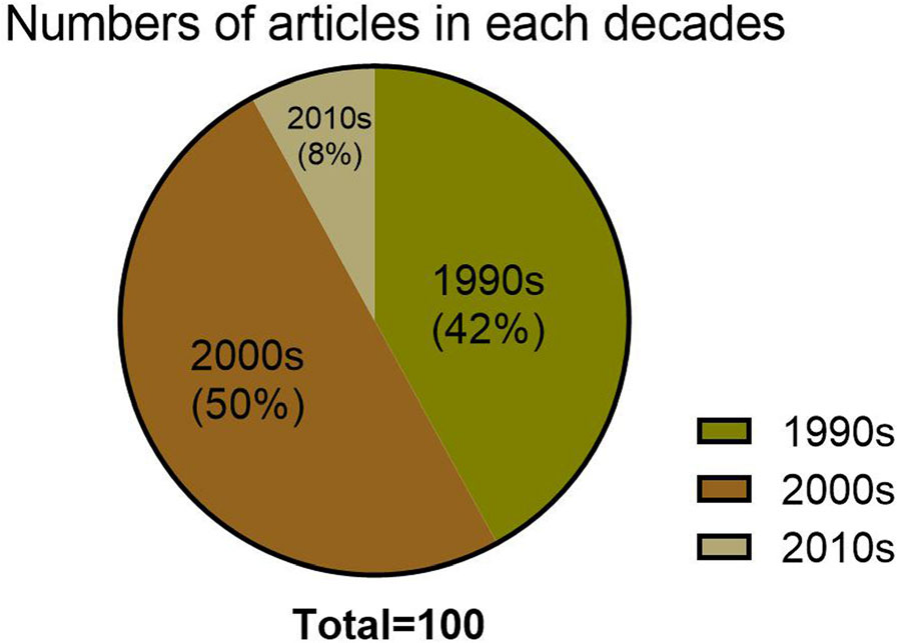
Time distribution of 100 top-cited articles in bronchoscopy. A majority of articles were published in the 1990s (42%, *n* = 42) and 2000s (50%, *n* = 50)

**Fig. 3:**
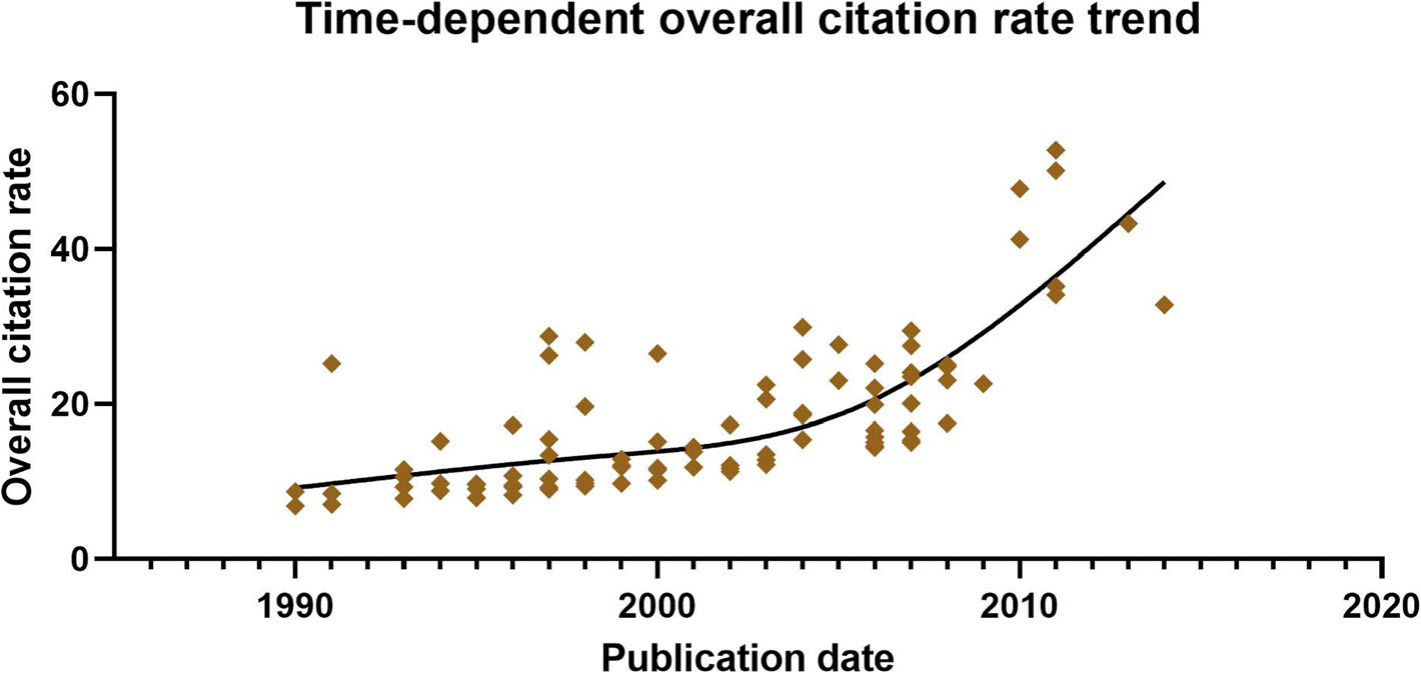
Time-dependent citation density trend. Mann-Kendall trend test showed an increasing trend between the citation density and the time (*p* < 0.0001)

We gathered the 100 most cited articles on bronchoscopy (Table 1*,* Additional file 1). The number of citations ranged from 731 to 196, and a majority of articles presented in the 2000s (50%) and 1990s (42%), while articles published in 2010s only accounted for 8% (Fig. 2). The year with the highest number of articles was 2006 (*n* = 9), followed by 2007 (*n* = 8), 1995 (*n* = 7), and 1997 (*n* = 7). The mean number of citations was 290 overall, 293 in the 1990s, 276 in the 2000s, and 368 in the 2010s. The mean number of citation density was 17.5 overall, 12.0 in the 1990s, 18.0 in the 2000s, and 42.2 in the 2010s. The Mann-Kendall trend test showed an increasing trend between the top-cited articles’ citation density and their published time (*P* < 0.0001) (Fig. 3). The Spearman rank showed a positive correlation between year of publication and citation density (*r* = 0.764, *P* < 0.01). Both the Shapiro-Wilk test and the Kolmogorov-Smirnov test indicated an abnormal distribution in total citation number as well as the citation density.

**Table 1:** List of the 100 top-cited articles on bronchoscopy

Rank	Article Title	Citations	overall citation rate
1	Pugin, J., et al., Diagnosis of ventilator-associated pneumonia by bacteriologic analysis of bronchoscopic and nonbronchoscopic “blind” bronchoalveolar lavage fluid. The American review of respiratory disease, 1991. 143(5 Pt 1): p. 1121–9. doi:10.1164/ajrccm/143.5_Pt_1.1121	731	25.2
2	Luna, C.M., et al., Impact of BAL data on the therapy and outcome of ventilator-associated pneumonia. Chest, 1997. 111(3): p. 676–85. doi:10.1378/chest.111.3.676	662	28.8
3	Trouillet, J.L., et al., Ventilator-associated pneumonia caused by potentially drug-resistant bacteria. American journal of respiratory and critical care medicine, 1998. 157(2): p. 531–9. doi:10.1164/ajrccm.157.2.9705064	615	28.0
4	Wenzel, S.E., et al., Bronchoscopic evaluation of severe asthma. Persistent inflammation associated with high dose glucocorticoids. American journal of respiratory and critical care medicine, 1997. 156(3 Pt 1): p. 737–43. doi:10.1164/ajrccm.156.3.9610046	604	26.3
5	Fagon, J.Y., et al., Invasive and noninvasive strategies for management of suspected ventilator-associated pneumonia - A randomized trial. Annals of Internal Medicine, 2000. 132(8): p. 621 − +. doi:10.7326/0003-4819-132-8-200,004,180-00004	530	26.5
6	Yasufuku, K., et al., Real-time endobronchial ultrasound-guided transbronchial needle aspiration of mediastinal and hilar lymph nodes. Chest, 2004. 126(1): p. 122–128. doi:10.1378/chest.126.1.122	479	29.9
7	Sciurba, F.C., et al., A Randomized Study of Endobronchial Valves for Advanced Emphysema. New England Journal of Medicine, 2010. 363(13): p. 1233–1244. doi:10.1056/NEJMoa0900928	478	47.8
8	Charlson, E.S., et al., Topographical Continuity of Bacterial Populations in the Healthy Human Respiratory Tract. American Journal of Respiratory and Critical Care Medicine, 2011. 184(8): p. 957–963. doi:10.1164/rccm.201104-0655OC	475	52.8
9	Erb-Downward, J.R., et al., Analysis of the Lung Microbiome in the “Healthy” Smoker and in COPD. Plos One, 2011. 6(2). doi:10.1371/journal.pone.0016384	451	50.1
10	Lam, S., et al., Localization of bronchial intraepithelial neoplastic lesions by fluorescence bronchoscopy. Chest, 1998. 113(3): p. 696–702. doi:10.1378/chest.113.3.696	433	19.7
11	Howarth, P.H., et al., Tumour necrosis factor (TNF alpha) as a novel therapeutic target in symptomatic corticosteroid dependent asthma. Thorax, 2005. 60(12): p. 1012–1018. doi:10.1136/thx.2005.045260	415	27.7
12	Kraft, M., et al., Alveolar tissue inflammation in asthma. American journal of respiratory and critical care medicine, 1996. 154(5): p. 1505–10. doi:10.1164/ajrccm.154.5.8912772	415	17.3
13	Castro, M., et al., Effectiveness and Safety of Bronchial Thermoplasty in the Treatment of Severe Asthma A Multicenter, Randomized, Double-Blind, Sham-Controlled Clinical Trial. American Journal of Respiratory and Critical Care Medicine, 2010. 181(2): p. 116–124. doi:10.1164/rccm.200903-0354OC	412	41.2
14	Spira, A., et al., Effects of cigarette smoke on the human airway epithelial cell transcriptome. Proceedings of the National Academy of Sciences of the United States of America, 2004. 101(27): p. 10143–10,148. doi:10.1073/pnas.0401422101	412	25.8
15	Saetta, M., et al., Airway eosinophilia in chronic bronchitis during exacerbations. American journal of respiratory and critical care medicine, 1994. 150(6 Pt 1): p. 1646–52. doi:10.1164/ajrccm.150.6.7952628	396	15.2
16	Cox, G., et al., Asthma control during the year after bronchial thermoplasty. New England Journal of Medicine, 2007. 356(13): p. 1327–1337. doi:10.1056/NEJMoa064707	383	29.5
17	Phillips, M., et al., Detection of lung cancer with volatile markers in the breath. Chest, 2003. 123(6): p. 2115–2123. doi:10.1378/chest.123.6.2115	382	22.5
18	Spira, A., et al., Airway epithelial gene expression in the diagnostic evaluation of smokers with suspect lung cancer. Nature Medicine, 2007. 13(3): p. 361–366. doi:10.1038/nm1556	358	27.5
19	Rello, J., et al., The value of routine microbial investigation in ventilator-associated pneumonia. American journal of respiratory and critical care medicine, 1997. 156(1): p. 196–200. doi:10.1164/ajrccm.156.1.9607030	356	15.5
20	Yasufuku, K., et al., Comparison of endobronchial ultrasound, positron emission tomography, and CT for lymph node staging of lung cancer. Chest, 2006. 130(3): p. 710–718. doi:10.1378/chest.130.3.710	353	25.2
21	Luna, C.M., et al., Resolution of ventilator-associated pneumonia: Prospective evaluation of the clinical pulmonary infection score as an early clinical predictor of outcome. Critical Care Medicine, 2003. 31(3): p. 676–682. doi:10.1097/01.ccm.0000055380.86458.1e	351	20.6
22	Yasufuku, K., et al., Endobronchial ultrasound guided transbronchial needle aspiration for staging of lung cancer. Lung Cancer, 2005. 50(3): p. 347–354. doi:10.1016/j.lungcan.2005.07.013	345	23.0
23	Grainge, C.L., et al., Effect of Bronchoconstriction on Airway Remodeling in Asthma. New England Journal of Medicine, 2011. 364(21): p. 2006–2015. doi:10.1056/NEJMoa1014350	317	35.2
24	Eberhardt, R., et al., Multimodality bronchoscopic diagnosis of peripheral lung lesions - A randomized controlled trial. American Journal of Respiratory and Critical Care Medicine, 2007. 176(1): p. 36–41. doi:10.1164/rccm.200612-1866OC	313	24.1
25	Lam, S., et al., Detection of dysplasia and carcinoma in situ with a lung imaging fluorescence endoscope device. The Journal of thoracic and cardiovascular surgery, 1993. 105(6): p. 1035–40. doi:10.1016/s0022-5223(19)33775-4	313	11.6
26	Ward, C., et al., Airway inflammation, basement membrane thickening and bronchial hyperresponsiveness in asthma. Thorax, 2002. 57(4): p. 309–316. doi:10.1136/thorax.57.4.309	312	17.3
27	Heyland, D., et al., A randomized trial of diagnostic techniques for ventilator-associated pneumonia. New England Journal of Medicine, 2006. 355(25): p. 2619–2630. doi:10.1056/NEJMoa052904	309	22.1
28	Li, X. and J.W. Wilson, Increased vascularity of the bronchial mucosa in mild asthma. American journal of respiratory and critical care medicine, 1997. 156(1): p. 229–33. doi:10.1164/ajrccm.156.1.9607066	309	13.4
29	Yasufuku, K., et al., A prospective controlled trial of endobronchial ultrasound-guided transbronchial needle aspiration compared with mediastinoscopy for mediastinal lymph node staging of lung cancer. Journal of Thoracic and Cardiovascular Surgery, 2011. 142(6): p. 1393–1402. doi:10.1016/j.jtcvs.2011.08.037	307	34.1
30	Saglani, S., et al., Early detection of airway wall remodeling and eosinophilic inflammation in preschool wheezers. American Journal of Respiratory and Critical Care Medicine, 2007. 176(9): p. 858–864. doi:10.1164/rccm.200702-212OC	306	23.5
31	Morris, A., et al., Comparison of the Respiratory Microbiome in Healthy Nonsmokers and Smokers. American Journal of Respiratory and Critical Care Medicine, 2013. 187(10): p. 1067–1075. doi:10.1164/rccm.201210-1913OC	303	43.3
32	Baaklini, W.A., et al., Diagnostic yield of fiberoptic bronchoscopy in evaluating solitary pulmonary nodules. Chest, 2000. 117(4): p. 1049–1054. doi:10.1378/chest.117.4.1049	303	15.2
33	Meersseman, W., et al., Galactomannan in bronchoalveolar lavage fluid - A tool for diagnosing aspergillosis in intensive care unit patients. American Journal of Respiratory and Critical Care Medicine, 2008. 177(1): p. 27–34. doi:10.1164/rccm.200704-606OC	301	25.1
34	Herth, F., H.D. Becker, and A. Ernst, Conventional vs endobronchial ultrasound-guided transbronchial needle aspiration - A randomized trial. Chest, 2004. 125(1): p. 322–325. doi:10.1378/chest.125.1.322	301	18.8
35	Woodruff, P.G., et al., Hyperplasia of smooth muscle in mild to moderate asthma without changes in cell size or gene expression. American Journal of Respiratory and Critical Care Medicine, 2004. 169(9): p. 1001–1006. doi:10.1164/rccm.200311-1529OC	299	18.7
36	Wallace, M.B., et al., Minimally invasive endoscopic staging of suspected lung cancer. Jama-Journal of the American Medical Association, 2008. 299(5): p. 540–546. doi:10.1001/jama.299.5.540	298	24.8
37	Kurimoto, N., et al., Endobronchial ultrasonography using a guide sheath increases the ability to diagnose peripheral pulmonary lesions endoscopically. Chest, 2004. 126(3): p. 959–965. doi:10.1378/chest.126.3.959	297	18.6
38	Roum, J.H., et al., Systemic deficiency of glutathione in cystic fibrosis. Journal of applied physiology (Bethesda, Md.: 1985), 1993. 75(6): p. 2419–24. doi:10.1152/jappl.1993.75.6.2419	287	10.6
39	Gildea, T.R., et al., Electromagnetic navigation diagnostic bronchoscopy - A prospective study. American Journal of Respiratory and Critical Care Medicine, 2006. 174(9): p. 982–989. doi:10.1164/rccm.200603-344OC	279	19.9
40	Makrygiannakis, D., et al., Smoking increases peptidylarginine deiminase 2 enzyme expression in human lungs and increases citrullination in BAL cells. Annals of the Rheumatic Diseases, 2008. 67(10): p. 1488–1492. doi:10.1136/ard.2007.075192	277	23.1
41	Takizawa, H., et al., Increased expression of transforming growth factor-beta 1 in small airway epithelium from tobacco smokers and patients with chronic obstructive pulmonary disease (COPD). American Journal of Respiratory and Critical Care Medicine, 2001. 163(6): p. 1476–1483. doi:10.1164/ajrccm.163.6.9908135	275	14.5
42	Muhlebach, M.S., et al., Quantitation of inflammatory responses to bacteria in young cystic fibrosis and control patients. American Journal of Respiratory and Critical Care Medicine, 1999. 160(1): p. 186–191. doi:10.1164/ajrccm.160.1.9808096	271	12.9
43	Rosenfeld, M., et al., Early pulmonary infection, inflammation, and clinical outcomes in infants with cystic fibrosis. Pediatric Pulmonology, 2001. 32(5): p. 356–366. doi:10.1002/ppul.1144	264	13.9
44	Limper, A.H. and U.B. Prakash, Tracheobronchial foreign bodies in adults. Annals of internal medicine, 1990. 112(8): p. 604–9. doi:10.7326/0003-4819-112-8-604	262	8.7
45	Pavord, I.D., et al., Safety and efficacy of bronchial thermoplasty in symptomatic, severe asthma. American Journal of Respiratory and Critical Care Medicine, 2007. 176(12): p. 1185–1191. doi:10.1164/rccm.200704-571OC	261	20.1
46	Schwartz, M.D., et al., Nuclear factor-kappa B is activated in alveolar macrophages from patients with acute respiratory distress syndrome. Critical care medicine, 1996. 24(8): p. 1285–92. doi:10.1097/00003246-199,608,000-00004	258	10.8
47	Soler, N., et al., Airway inflammation and bronchial microbial patterns in patients with stable chronic obstructive pulmonary disease. European Respiratory Journal, 1999. 14(5): p. 1015–1022. doi:10.1183/09031936.99.14510159	255	12.1
48	Trigg, C.J., et al., Placebo-controlled immunopathologic study of four months of inhaled corticosteroids in asthma. American journal of respiratory and critical care medicine, 1994. 150(1): p. 17–22. doi:10.1164/ajrccm.150.1.8025745	254	9.8
49	Steinberg, K.P., et al., Evolution of bronchoalveolar cell populations in the adult respiratory distress syndrome. American journal of respiratory and critical care medicine, 1994. 150(1): p. 113–22. doi:10.1164/ajrccm.150.1.8025736	254	9.8
50	Driver, A.G., et al., Adenosine in bronchoalveolar lavage fluid in asthma. The American review of respiratory disease, 1993. 148(1): p. 91–7. doi:10.1164/ajrccm/148.1.91	252	9.3
51	Baharloo, F., et al., Tracheobronchial foreign bodies - Presentation and management in children and adults. Chest, 1999. 115(5): p. 1357–1362. doi:10.1378/chest.115.5.1357	250	11.9
52	Tremblay, A., et al., A Randomized Controlled Trial of Standard vs Endobronchial Ultrasonography-Guided Transbronchial Needle Aspiration in Patients With Suspected Sarcoidosis. Chest, 2009. 136(2): p. 340–346. doi:10.1378/chest.08-2768	249	22.6
53	Groneberg, D.A., et al., Increased expression of transient receptor potential vanilloid-1 in airway nerves of chronic cough. American Journal of Respiratory and Critical Care Medicine, 2004. 170(12): p. 1276–1280. doi:10.1164/rccm.200402-174OC	247	15.4
54	Martin, R.J., et al., Airways inflammation in nocturnal asthma. The American review of respiratory disease, 1991. 143(2): p. 351–7. doi:10.1164/ajrccm/143.2.351	246	8.5
55	Balough, K., et al., The relationship between infection and inflammation in the early stages of lung disease from cystic fibrosis. Pediatric pulmonology, 1995. 20(2): p. 63–70. doi:10.1002/ppul.1950200203	243	9.7
56	Marquette, C.H., et al., Diagnostic tests for pneumonia in ventilated patients: prospective evaluation of diagnostic accuracy using histology as a diagnostic gold standard. American journal of respiratory and critical care medicine, 1995. 151(6): p. 1878–88. doi:10.1164/ajrccm.151.6.7767535	241	9.6
57	Papazian, L., et al., Bronchoscopic or blind sampling techniques for the diagnosis of ventilator-associated pneumonia. American journal of respiratory and critical care medicine, 1995. 152(6 Pt 1): p. 1982–91. doi:10.1164/ajrccm.152.6.8520766	240	9.6
58	Olivieri, D., et al., Effect of short-term treatment with low-dose inhaled fluticasone propionate on airway inflammation and remodeling in mild asthma: a placebo-controlled study. American journal of respiratory and critical care medicine, 1997. 155(6): p. 1864–71. doi:10.1164/ajrccm.155.6.9196087	239	10.4
59	Chastre, J., et al., Evaluation of bronchoscopic techniques for the diagnosis of nosocomial pneumonia. American journal of respiratory and critical care medicine, 1995. 152(1): p. 231–40. doi:10.1164/ajrccm.152.1.7599829	239	9.6
60	Rutgers, S.R., et al., Ongoing airway inflammation in patients with COPD who do not currently smoke. Thorax, 2000. 55(1): p. 12–18. doi:10.1136/thorax.55.1.12	235	11.8
61	von Eiff, M., et al., Pulmonary aspergillosis: early diagnosis improves survival. Respiration; international review of thoracic diseases, 1995. 62(6): p. 341–7. doi:10.1159/000196477	235	9.4
62	Marchant, J.M., et al., Evaluation and outcome of young children with chronic cough. Chest, 2006. 129(5): p. 1132–1141. doi:10.1378/chest.129.5.1132	233	16.6
63	Qiu, Y.S., et al., Biopsy neutrophilia, neutrophil chemokine and receptor gene expression in severe exacerbations of chronic obstructive pulmonary disease. American Journal of Respiratory and Critical Care Medicine, 2003. 168(8): p. 968–975. doi:10.1164/rccm.200208-794OC	230	13.5
64	Salvi, S.S., et al., Acute exposure to diesel exhaust increases IL-8 and GRO-alpha production in healthy human airways. American Journal of Respiratory and Critical Care Medicine, 2000. 161(2): p. 550–557. doi:10.1164/ajrccm.161.2.9905052	230	11.5
65	Utz, J.P., S.J. Swensen, and M.A. Gertz, Pulmonary amyloidosis. The Mayo Clinic experience from 1980 to 1993. Annals of internal medicine, 1996. 124(4): p. 407–13. doi:10.7326/0003-4819-124-4-199,602,150-00004	229	9.5
66	Calhoun, W.J., et al., A common cold virus, rhinovirus 16, potentiates airway inflammation after segmental antigen bronchoprovocation in allergic subjects. The Journal of clinical investigation, 1994. 94(6): p. 2200–8. doi:10.1172/jci117581	229	8.8
67	Fahy, J.V., et al., Comparison of samples collected by sputum induction and bronchoscopy from asthmatic and healthy subjects. American journal of respiratory and critical care medicine, 1995. 152(1): p. 53–8. doi:10.1164/ajrccm.152.1.7599862	227	9.1
68	Morelon, E., et al., Characteristics of sirolimus-associated interstitial pneumonitis in renal transplant patients. Transplantation, 2001. 72(5): p. 787–790. doi:10.1097/00007890-200,109,150-00008	226	11.9
69	Cavaliere, S., et al., Endoscopic treatment of malignant airway obstructions in 2008 patients. Chest, 1996. 110(6): p. 1536–42. doi:10.1378/chest.110.6.1536	226	9.4
70	Sanchez-Nieto, J.M., et al., Impact of invasive and noninvasive quantitative culture sampling on outcome of ventilator-associated pneumonia: a pilot study. American journal of respiratory and critical care medicine, 1998. 157(2): p. 371–6. doi:10.1164/ajrccm.157.2.97-02039	225	10.2
71	Vining, D.J., et al., Virtual bronchoscopy. Relationships of virtual reality endobronchial simulations to actual bronchoscopic findings. Chest, 1996. 109(2): p. 549–53. doi:10.1378/chest.109.2.549	224	9.3
72	Sethi, S., et al., Airway inflammation and bronchial bacterial colonization in chronic obstructive pulmonary disease. American Journal of Respiratory and Critical Care Medicine, 2006. 173(9): p. 991–998. doi:10.1164/rccm.200509-1525OC	221	15.8
73	Toma, T.P., et al., Bronchoscopic volume reduction with valve implants in patients with severe emphysema. Lancet, 2003. 361(9361): p. 931–933. doi:10.1016/s0140-6736(03)12762-6	218	12.8
74	Swenson, E.R., et al., Pathogenesis of high-altitude pulmonary edema - Inflammation is not an etiologic factor. Jama-Journal of the American Medical Association, 2002. 287(17): p. 2228–2235. doi:10.1001/jama.287.17.2228	218	12.1
75	Pesci, A., et al., Inflammatory cells and mediators in bronchial lavage of patients with chronic obstructive pulmonary disease. The European respiratory journal, 1998. 12(2): p. 380–6. doi:10.1183/09031936.98.12020380	215	9.8
76	Thiberville, L., et al., In vivo imaging of the bronchial wall microstructure using fibered confocal fluorescence microscopy. American Journal of Respiratory and Critical Care Medicine, 2007. 175(1): p. 22–31. doi:10.1164/rccm.200605-684OC	214	16.5
77	Hirshberg, B., et al., Hemoptysis: etiology, evaluation, and outcome in a tertiary referral hospital. Chest, 1997. 112(2): p. 440–4. doi:10.1378/chest.112.2.440	213	9.3
78	Schwarz, Y., et al., Real-time electromagnetic navigation bronchoscopy to peripheral lung lesions using overlaid CT images - The first human study. Chest, 2006. 129(4): p. 988–994. doi:10.1378/chest.129.4.988	211	15.1
79	Conte, J.E., et al., Intrapulmonary pharmacokinetics of linezolid. Antimicrobial Agents and Chemotherapy, 2002. 46(5): p. 1475–1480. doi:10.1128/aac.46.5.1475-1480.2002	211	11.7
80	Lamer, C., et al., Analysis of vancomycin entry into pulmonary lining fluid by bronchoalveolar lavage in critically ill patients. Antimicrobial agents and chemotherapy, 1993. 37(2): p. 281–6. doi:10.1128/aac.37.2.281	211	7.8
81	Lee, H.S., et al., Real-time endobronchial ultrasound-guided transbronchial needle aspiration in mediastinal staging of non-small cell lung cancer - How many aspirations per target lymph node station? Chest, 2008. 134(2): p. 368–374. doi:10.1378/chest.07-2105	210	17.5
82	Diette, G.B., et al., Distraction therapy with nature sights and sounds reduces pain during flexible bronchoscopy - A complementary approach to routine analgesia. Chest, 2003. 123(3): p. 941–948. doi:10.1378/chest.123.3.941	208	12.2
83	Warke, T.J., et al., Exhaled nitric oxide correlates with airway eosinophils in childhood asthma. Thorax, 2002. 57(5): p. 383–387. doi:10.1136/thorax.57.5.383	207	11.5
84	Virchow, J.C., et al., Neurotrophins are increased in bronchoalveolar lavage fluid after segmental allergen provocation. American journal of respiratory and critical care medicine, 1998. 158(6): p. 2002–5. doi:10.1164/ajrccm.158.6.9803023	207	9.4
85	Gizycki, M.J., et al., Myofibroblast involvement in the allergen-induced late response in mild atopic asthma. American journal of respiratory cell and molecular biology, 1997. 16(6): p. 664–73. doi:10.1165/ajrcmb.16.6.9191468	207	9.0
86	Fagon, J.Y., et al., Characterization of distal bronchial microflora during acute exacerbation of chronic bronchitis. Use of the protected specimen brush technique in 54 mechanically ventilated patients. The American review of respiratory disease, 1990. 142(5): p. 1004–8. doi:10.1164/ajrccm/142.5.1004	206	6.9
87	Barbato, A., et al., Epithelial damage and angiogenesis in the airways of children with asthma. American Journal of Respiratory and Critical Care Medicine, 2006. 174(9): p. 975–981. doi:10.1164/rccm.200602-189OC	205	14.6
88	Morrison, D., et al., Epithelial permeability, inflammation, and oxidant stress in the air spaces of smokers. American Journal of Respiratory and Critical Care Medicine, 1999. 159(2): p. 473–479. doi:10.1164/ajrccm.159.2.9804080	205	9.8
89	Pham, L.H., et al., Diagnosis of nosocomial pneumonia in mechanically ventilated patients. Comparison of a plugged telescoping catheter with the protected specimen brush. The American review of respiratory disease, 1991. 143(5 Pt 1): p. 1055–61. doi:10.1164/ajrccm/143.5_Pt_1.1055	205	7.1
90	Angrill, J., et al., Bacterial colonisation in patients with bronchiectasis: microbiological pattern and risk factors. Thorax, 2002. 57(1): p. 15–19. doi:10.1136/thorax.57.1.15	204	11.3
91	Ruiz, M., et al., Noninvasive versus invasive microbial investigation in ventilator-associated pneumonia - Evaluation of outcome. American Journal of Respiratory and Critical Care Medicine, 2000. 162(1): p. 119–125. doi:10.1164/ajrccm.162.1.9907090	204	10.2
92	Hattotuwa, K.L., et al., The effects of inhaled fluticasone on airway inflammation in chronic obstructive pulmonary disease - A double-blind, placebo-controlled biopsy study. American Journal of Respiratory and Critical Care Medicine, 2002. 165(12): p. 1592–1596. doi:10.1164/rccm.2105025	203	11.3
93	Verleden, G.M., et al., Azithromycin reduces airway neutrophilia and interleukin-8 in patients with bronchiolitis obliterans syndrome. American Journal of Respiratory and Critical Care Medicine, 2006. 174(5): p. 566–570. doi:10.1164/rccm.200601-071OC	202	14.4
94	Cox, G., et al., Bronchial thermoplasty for asthma. American Journal of Respiratory and Critical Care Medicine, 2006. 173(9): p. 965–969. doi:10.1164/rccm.200507-1162OC	201	14.4
95	Harrow, E.M., et al., The utility of transbronchial needle aspiration in the staging of bronchogenic carcinoma. American Journal of Respiratory and Critical Care Medicine, 2000. 161(2): p. 601–607. doi:10.1164/ajrccm.161.2.9902040	201	10.1
96	Garwood, S., et al., Endobronchial ultrasound for the diagnosis of pulmonary sarcoidosis. Chest, 2007. 132(4): p. 1298–1304. doi:10.1378/chest.07-0998	200	15.4
97	Armstrong, D.S., et al., Bronchoalveolar lavage or oropharyngeal cultures to identify lower respiratory pathogens in infants with cystic fibrosis. Pediatric pulmonology, 1996. 21(5): p. 267–75. doi:10.1002/(sici)1099-0496(199605)21:5 < 267::aid-ppul1 > 3.0.co;2-k	198	8.3
98	Pue, C.A. and E.R. Pacht, Complications of fiberoptic bronchoscopy at a university hospital. Chest, 1995. 107(2): p. 430–2. doi:10.1378/chest.107.2.430	198	7.9
99	Molyneaux, P.L., et al., The Role of Bacteria in the Pathogenesis and Progression of Idiopathic Pulmonary Fibrosis. American Journal of Respiratory and Critical Care Medicine, 2014. 190(8): p. 906–913. doi:10.1164/rccm.201403-0541OC	197	32.8
100	Eberhardt, R., et al., Electromagnetic navigation diagnostic bronchoscopy in peripheral lung lesions. Chest, 2007. 131(6): p. 1800–1805. doi:10.1378/chest.06-3016	196	15.1

**Fig. 4:**
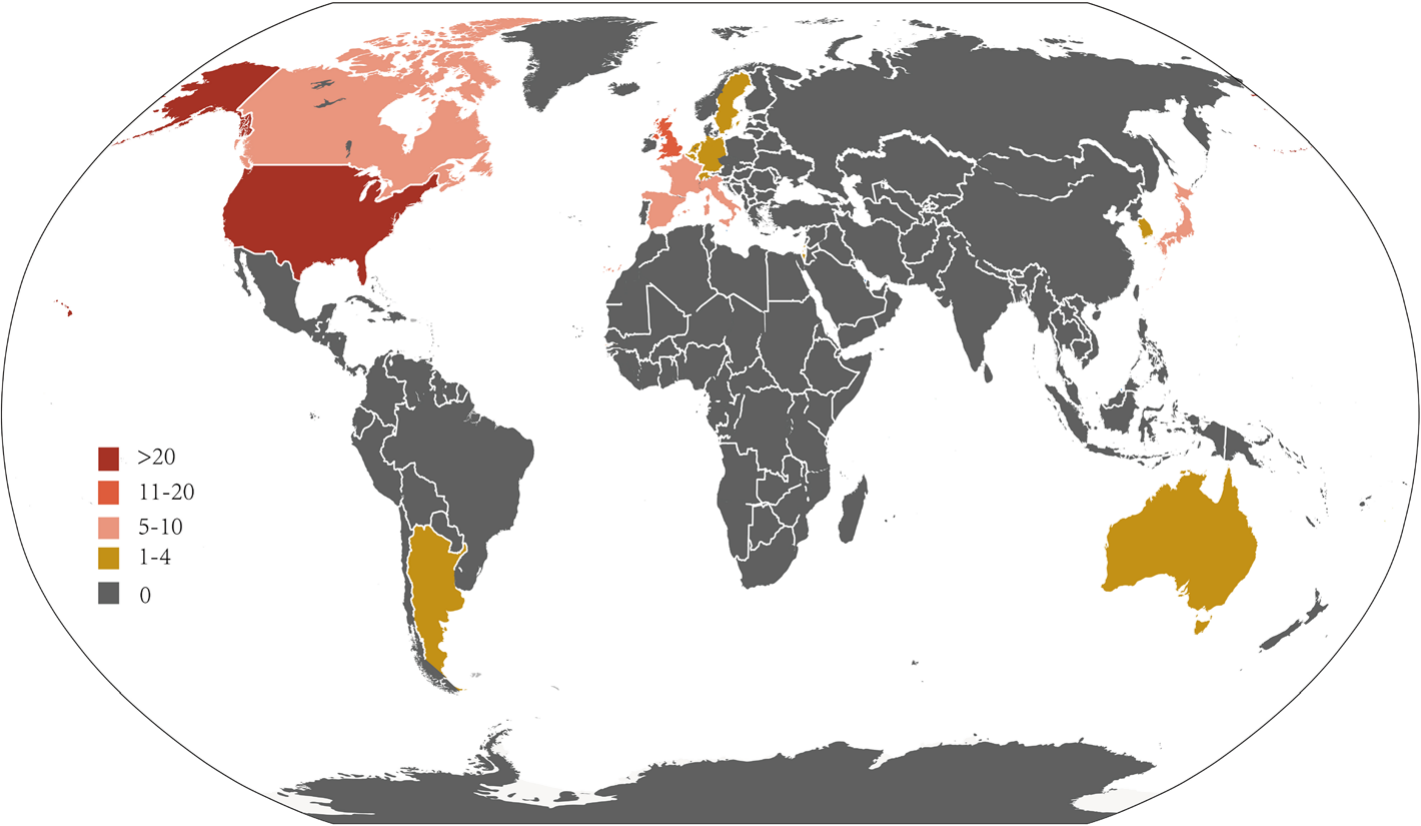
Geographical distribution of all articles. The map showed that most articles came from two regions: North America and Western Europe (Due to limited space, the national borders are not completely accurate. This figure was modified based on an original image downloaded from Wikimedia Commons https://upload.wikimedia.org/wikipedia/commons/e/e7/GDP_per_capita-worldmap-2006.svg)

All of the articles were distributed in 16 countries, led by the USA (*n* = 35), followed by the United Kingdom (*n* = 14), France (*n* = 10), and so on. The distribution was illustrated on the world map (Fig. 4). The map showed that two regions, North America and Western Europe, kept most of the articles. Besides, Japan had 6 articles, Australia had 4, Argentina and Israel both had 2, while Korea had 1.

These articles were published in 25 kinds of journals. Thirty-nine articles were published in *American journal of respiratory and critical care medicine*, followed by *Chest* (*n* = 20), *The American review of respiratory disease* (*n* = 5), *Thorax* (*n* = 5), *New England Journal of Medicine* (*n* = 4), *Annals of Internal Medicine* (*n* = 3), *Pediatric Pulmonology* (*n* = 3), and other three journals (*n* = 2) (Table 2).

**Table 2:** Journals with the most occurrences within the top-cited list and their impact factor (IF)

Journal name	No. of articles	IF (2019)
American journal of respiratory and critical care medicine	39	17.452
Chest	20	8.308
The American review of respiratory disease	5	17.452
Thorax	5	8.834
New England Journal of Medicine	4	74.699
Annals of Internal Medicine	3	21.317
Pediatric Pulmonology	3	2.534
Critical Care Medicine	2	7.414
Jama-Journal of the American Medical Association	2	45.54
Antimicrobial Agents and Chemotherapy	2	4.904

The first authors with the most occurrences and their basic research institutions were listed in Table 3. Yasufuku K, from Japan, had acted as the first authorship in four articles out of our list, in which two written in Chiba University, one in Department Hospital de Sabadell, and one in University of Toronto. The total citations of his four selected articles were 1484.

**Table 3:** List of first authors with the most occurrences within the top-cited list

First author	No. of articles	Affiliation
Yasufuku K	4	Chiba University;Department Hospital de Sabadell;University of Toronto
Cox G	2	McMaster University
Eberhardt R	2	Ruprecht Karls University Heidelberg;Harvard University
Fagon JY	2	Hopital Bichat
Lam S	2	British Columbia Cancer Research Center
Luna CM	2	University of Buenos Aires
Spira A	2	Boston University

We collected the research institutions of first authors in these top-cited articles. Eight institutions had 3 articles included, and they were Hospital Bichat, University of Barcelona, Royal Brompton Hospital, University of London, University of Southampton, Mayo Clinic, National Jewish Medical and Research Center, and University of California at San Francisco. Besides, 10 research institutions were involved by two articles listed, and 56 institutions with one article, respectively (Supplementary Table 1).

**Fig. 5:**
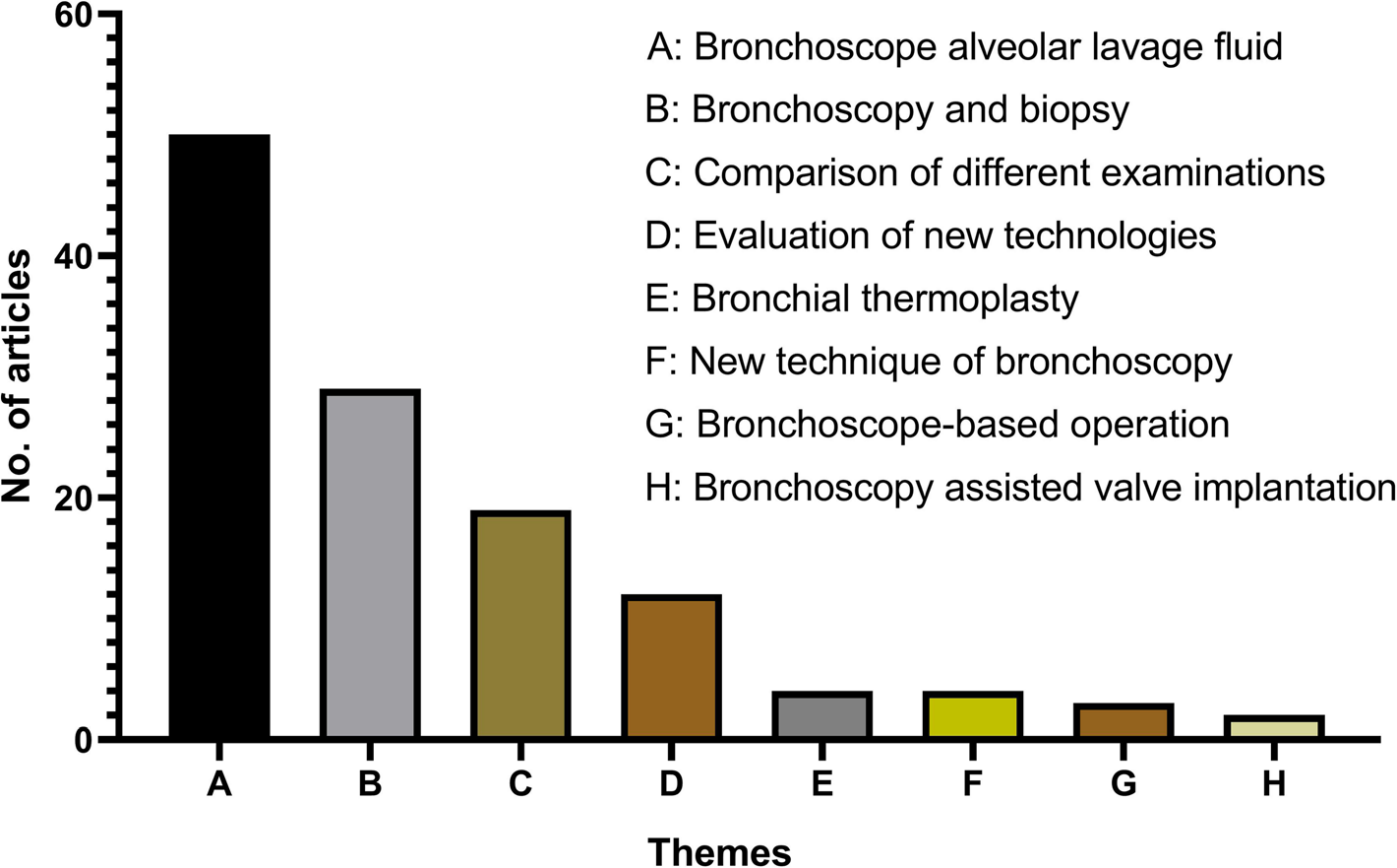
The themes distribution of all the articles. The most mentioned theme was bronchoalveolar lavage fluid (*n* = 50), followed by bronchoscopy and biopsy (*n* = 29)

**Fig. 6:**
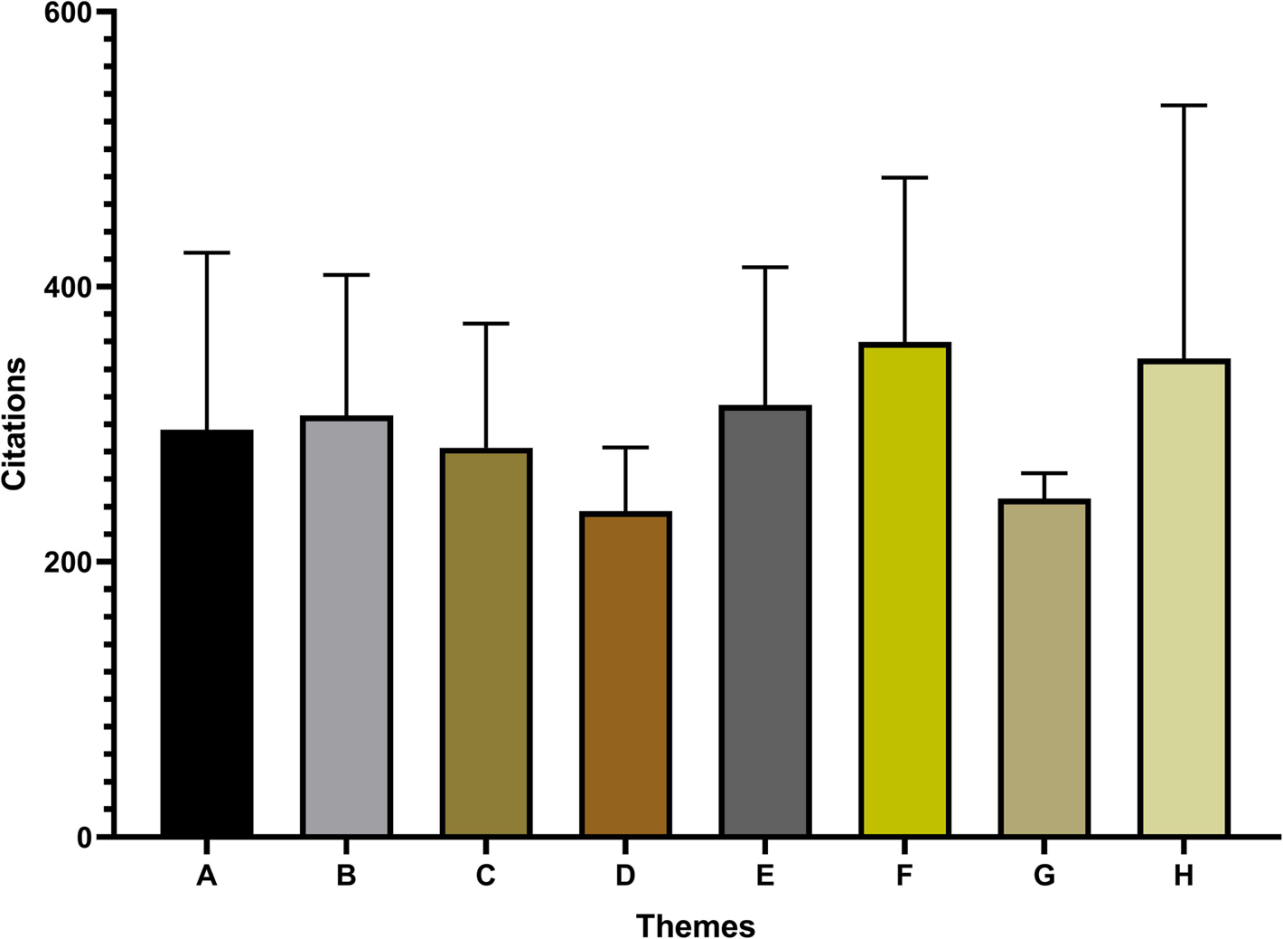
Mean citation per article based on the theme. One-way ANOVA showed no significant difference in citations per article among the various themes (*P* = 0.486). A: bronchoalveolar lavage fluid, B: bronchoscopy and biopsy, C: comparison of different examinations, D: evaluation of new technologies, E: bronchial thermoplasty, F: new technique of bronchoscopy, G: bronchoscope-based operation, H: bronchoscopy assisted valve implantation

We divided these top 100 most cited articles into eight categories based on the main content. In particular, some articles cover two or three types of topics at the same time, and we also recorded them repeatedly. Three professional researchers discussed each article and reached consensus. List these theme categories in descending order according to the number of relevant literature as: BAL fluid (*n* = 50), bronchoscopy and biopsy (*n* = 29), comparison of different examinations (*n* = 19), evaluation of new technologies (*n* = 12), bronchial thermoplasty (*n* = 4), new technique of bronchoscopy (*n* = 4), bronchoscope-based operation (*n* = 3), and bronchoscopy assisted valve implantation (*n* = 2) (Fig. 5). One-way ANOVA showed no significant difference between citations and these themes (*P* = 0.486) (Fig. 6).

**Fig. 7:**
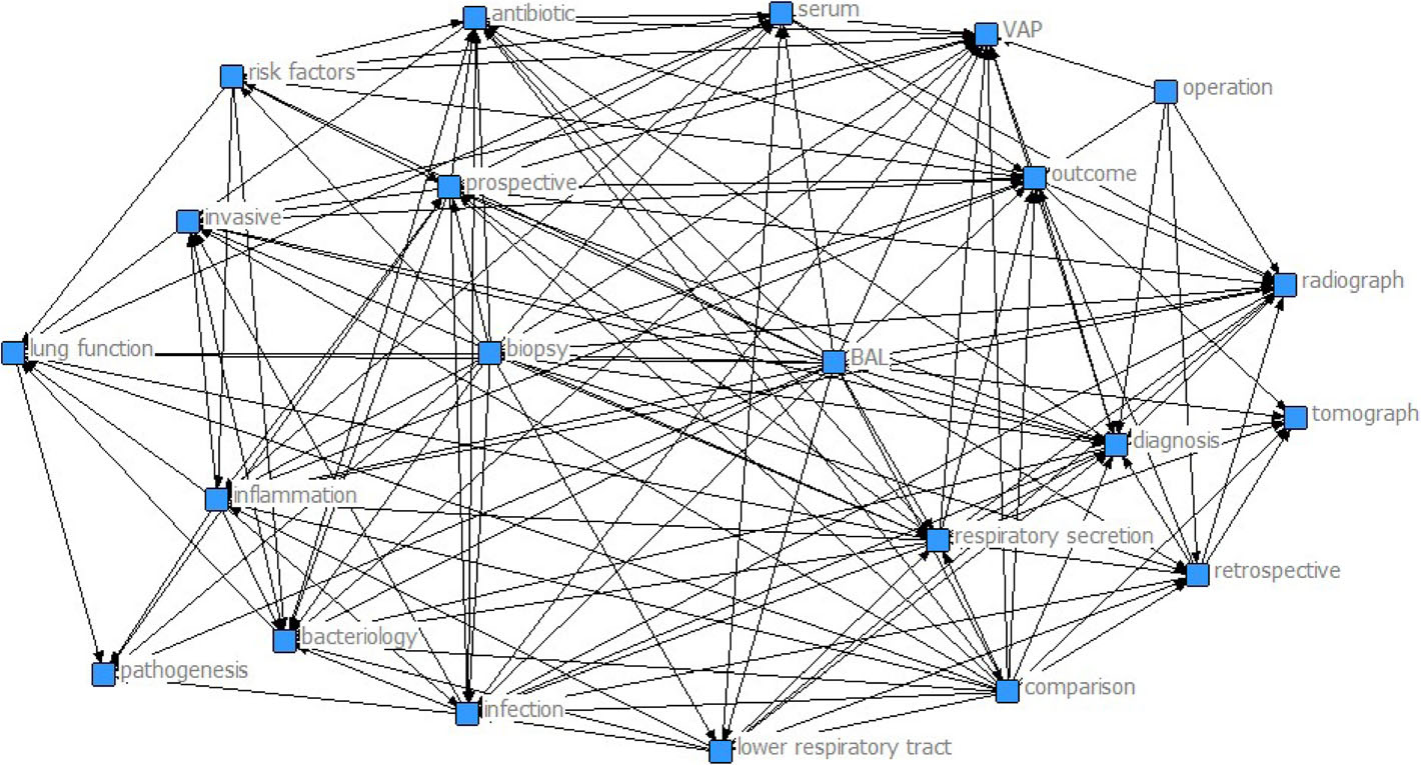
Degree centrality analysis in the 1990s (42 articles). It showed that “BAL, inflammation, diagnosis, biopsy” had a high degree of centrality in the 1990s

**Fig. 8:**
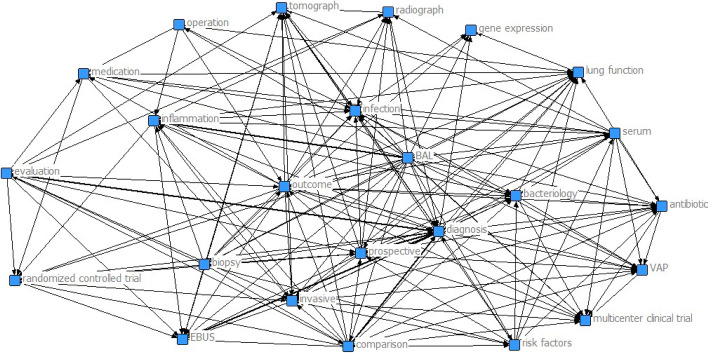
Degree centrality analysis in the 2000s (50 articles). It showed that “diagnosis, BAL, biopsy, prospective, outcome, EBUS, evaluation” had a high degree of centrality in the 2000s

These article’s keywords and subject terms were checked one by one by two researchers, and then degree centrality analysis has been done in two periods of article published time: in the 1990s (42 articles) and the 2000s (50 articles). The result indicated that “BAL, inflammation, diagnosis, biopsy” had a high degree of centrality in the 1990s (Fig. 7), while “diagnosis, BAL, biopsy, prospective, outcome, EBUS, evaluation” had a high degree of centrality in the 2000s (Fig. 8).

By ranking the Local Citation Score (LCS) index from high to low, we got the top 30 articles with the LCS of 46 to 13 in HistCite Pro 2.1(Table 4). We compared them with the top 100 articles selected by the former method and found that 12 of them appeared again.

**Table 4:** List of the top 30 local citation score (LCS) articles in top 1000 cited articles on bronchoscopy

Rank	Article Title	LCS
1^a^	Baaklini, W.A., et al., Diagnostic yield of fiberoptic bronchoscopy in evaluating solitary pulmonary nodules. Chest, 2000. 117(4): p. 1049–1054. doi: 10.1378/chest.117.4.1049	46
2	Schreiber, G. and D.C. McCrory, Performance characteristics of different modalities for diagnosis of suspected lung cancer - Summary of published evidence. Chest, 2003. 123(1): p. 115S–128S. doi: 10.1378/chest.123.1_suppl.115S	29
3^a^	Gildea, T.R., et al., Electromagnetic navigation diagnostic bronchoscopy - A prospective study. American Journal of Respiratory and Critical Care Medicine, 2006. 174(9): p. 982–989. doi: 10.1164/rccm.200603-344OC	28
4	Kurimoto, N., et al., Assessment of usefulness of endobronchial ultrasonography in determination of depth of tracheobronchial tumor invasion. Chest, 1999. 115(6): p. 1500–1506. doi: 10.1378/chest.115.6.1500	25
5^a^	Harrow, E.M., et al., The utility of transbronchial needle aspiration in the staging of bronchogenic carcinoma. American Journal of Respiratory and Critical Care Medicine, 2000. 161(2): p. 601–607. doi: 10.1164/ajrccm.161.2.9902040	23
6^a^	Eberhardt, R., et al., Multimodality bronchoscopic diagnosis of peripheral lung lesions - A randomized controlled trial. American Journal of Respiratory and Critical Care Medicine, 2007. 176(1): p. 36–41. doi: 10.1164/rccm.200612-1866OC	22
7^a^	Toma, T.P., et al., Bronchoscopic volume reduction with valve implants in patients with severe emphysema. Lancet, 2003. 361(9361): p. 931–933. doi: 10.1016/s0140-6736(03)12762-6	21
8	Herth, F.J.F., A. Ernst, and H.D. Becker, Endobronchial ultrasound-guided transbronchial lung biopsy in solitary pulmonary nodules and peripheral lesions. European Respiratory Journal, 2002. 20(4): p. 972–974. doi: 10.1183/09031936.02.00032001	20
9^a^	Schwarz, Y., et al., Real-time electromagnetic navigation bronchoscopy to peripheral lung lesions using overlaid CT images - The first human study. Chest, 2006. 129(4): p. 988–994. doi: 10.1378/chest.129.4.988	20
10^a^	Kurimoto, N., et al., Endobronchial ultrasonography using a guide sheath increases the ability to diagnose peripheral pulmonary lesions endoscopically. Chest, 2004. 126(3): p. 959–965. doi: 10.1378/chest.126.3.959	19
11^a^	Yasufuku, K., et al., Real-time endobronchial ultrasound-guided transbronchial needle aspiration of mediastinal and hilar lymph nodes. Chest, 2004. 126(1): p. 122–128. doi: 10.1378/chest.126.1.122	19
12	Venmans, B.J.W., et al., Outcome of bronchial carcinoma in situ. Chest, 2000. 117(6): p. 1572–1576. doi: 10.1378/chest.117.6.1572	18
13	Garpestad, E., et al., CT fluoroscopy guidance for transbronchial needle aspiration - An experience in 35 patients. Chest, 2001. 119(2): p. 329–332. doi: 10.1378/chest.119.2.329	18
14	Paone, G., et al., Endobronchial ultrasound-driven biopsy in the diagnosis of peripheral lung lesions. Chest, 2005. 128(5): p. 3551–3557. doi: 10.1378/chest.128.5.3551	18
15	Schwarz, Y., et al., Electromagnetic navigation during flexible bronchoscopy. Respiration, 2003. 70(5): p. 516–522. doi: 10.1159/000074210	17
16	Snell, G.I., et al., The potential for bronchoscopic lung volume reduction using bronchial prostheses - A pilot study. Chest, 2003. 124(3): p. 1073–1080. doi: 10.1378/chest.124.3.1073	17
17	Shinagawa, N., et al., CT-guided transbronchial biopsy using an ultrathin bronchoscope with virtual bronchoscopic navigation. Chest, 2004. 125(3): p. 1138–1143. doi: 10.1378/chest.125.3.1138	17
18	Haponik, E.F., S.L. Aquino, and D.J. Vining, Virtual bronchoscopy. Clinics in Chest Medicine, 1999. 20(1): p. 201 − +. doi: 10.1016/s0272-5231(05)70135-0	16
19	Hirsch, F.R., et al., Fluorescence versus white-light bronchoscopy for detection of preneoplastic lesions: a randomized study. Journal of the National Cancer Institute, 2001. 93(18): p. 1385–1391. doi: 10.1093/jnci/93.18.1385	16
20	Herth, F.J.F., et al., Endobronchial ultrasound-guided transbronchial lung biopsy in fluoroscopically invisible solitary pulmonary nodules - A prospective trial. Chest, 2006. 129(1): p. 147–150. doi: 10.1378/chest.129.1.147	16
21	Reichenberger, F., et al., The value of transbronchial needle aspiration in the diagnosis of peripheral pulmonary lesions. Chest, 1999. 116(3): p. 704–708. doi: 10.1378/chest.116.3.704	15
22	Hopkinson, N.S., et al., Effect of Bronchoscopic lung volume reduction on dynamic hyperinflation and exercise in emphysema. American Journal of Respiratory and Critical Care Medicine, 2005. 171(5): p. 453–460. doi: 10.1164/rccm.200407-961OC	15
23^a^	Eberhardt, R., et al., Electromagnetic navigation diagnostic bronchoscopy in peripheral lung lesions. Chest, 2007. 131(6): p. 1800–1805. doi: 10.1378/chest.06-3016	15
24	Bota, S., et al., Follow-up of bronchial precancerous lesions and carcinoma in situ using fluorescence endoscopy. American Journal of Respiratory and Critical Care Medicine, 2001. 164(9): p. 1688–1693. doi: 10.1164/ajrccm.164.9.2012147	14
25^a^	Herth, F., H.D. Becker, and A. Ernst, Conventional vs endobronchial ultrasound-guided transbronchial needle aspiration - A randomized trial. Chest, 2004. 125(1): p. 322–325. doi: 10.1378/chest.125.1.322	14
26	Kikuchi, E., et al., Endobronchial ultrasonography with guide-sheath for peripheral pulmonary lesions. European Respiratory Journal, 2004. 24(4): p. 533–537. doi: 10.1183/09031936.04.00138603	14
27	Shirakawa, T., et al., Usefulness of endobronchial ultrasonography for transbronchial lung biopsies of peripheral lung lesions. Respiration, 2004. 71(3): p. 260–268. doi: 10.1159/000077424	14
28	Asahina, H., et al., Transbronchial biopsy using endobronchial ultrasonography with a guide sheath and virtual bronchoscopic navigation. Chest, 2005. 128(3): p. 1761–1765. doi: 10.1378/chest.128.3.1761	14
29^a^	Yasufuku, K., et al., Endobronchial ultrasound guided transbronchial needle aspiration for staging of lung cancer. Lung Cancer, 2005. 50(3): p. 347–354. doi: 10.1016/j.lungcan.2005.07.013	14
30^a^	Fagon, J.Y., et al., Invasive and noninvasive strategies for management of suspected ventilator-associated pneumonia - A randomized trial. Annals of Internal Medicine, 2000. 132(8): p. 621 − +. doi: 10.7326/0003-4819-132-8-200,004,180-00004	13

^a^ The same articles appeared in Table 1

## Discussion

As far as we can know, this is the first bibliometric analysis of papers on bronchoscopy. From the analysis of the top 100 most cited articles published on the theme of bronchoscopy, we can get several significant findings. The maximum number of citations in these articles reached 731, and this was a paper based on a bacteriologic analysis of BAL fluid for the diagnosis of ventilator-associated pneumonia [9]. When it comes to such relatively limited citation numbers, one explanation is that our analysis only focuses on articles published in the last 30 years, so there may not be enough time for them to be fully cited. Half of the top-cited articles were published in the 2000s, seconded by the 1990s with the article number of 42 in the top 100 most cited. The phenomenon of “obliteration by incorporation”, which refers to articles no longer be cited when their substance has been treated as current knowledge [7], maybe exists in this analysis. Besides, there is an increasing trend between top-cited articles’ citation density and their published time, showing that more and more high-quality articles are published in this field. Interestingly, there are eight articles published since 2010 making the top 100 most cited. The most recent literature in them was published in 2014, with the citation of 197 now [10]; followed by an article in 2013, with the citation of 303 here [11]. These results above also suggest that quite a few highly cited articles about bronchoscopy have appeared in the past 10 years.

We also have demonstrated the regional distribution of these articles. On the one hand, the majority of them originated from two regions, North America and Western Europe. Many research institutions there also performed well. For example, eight institutions each contributed three articles in the top 100 most cited, three of them (Mayo Clinic, National Jewish Medical and Research Center, and the University of California at San Francisco) from the USA, three of them (University of Southampton, University of London, and Royal Brompton Hospital) from the United Kingdom, along with Hospital Bichat and University of Barcelona from France and Spain individually. The reason for this phenomenon may be countries such as the USA and the United Kingdom have a fairly developed economy for supporting medical research. And it has previously been illustrated that a weak correlation exists between a country’s gross domestic product per capita and its research achievements [12]. On the other hand, we can see that some papers are from Japan and Korea in Asia, the Republic of Argentina in South America, and Australia in Oceania, suggesting that the application of bronchoscopy is universal and proving that this inspection technique has always been of research value once again. It means a lot that the research achievements from all these countries are shared in public so that more people can benefit from their studies.

We noticed that two journals contributed more than a half articles in the top 100 most cited, demonstrating strong professional attributes. They are *American journal of respiratory and critical care medicine* (*AJRCCM*) with the Impact Factor of 17.452 now and *Chest* Journal, whose Impact Factor in 2019 is 8.308. In our analysis, there are 39 articles from *AJRCCM* with 6 kinds of theme categories, and 20 articles from *Chest* also got involved in almost all of the themes we cared about. Among them, 9 articles of *Chest* and 3 articles of *AJRCCM* talked about the evaluation of new technologies, making up all articles in such category. Unlike the phenomenon in a similar bibliometric analysis of respiratory articles [13], it seems several core journals here gleaned a lot of citations. Apart from those specific journals in this field, some general medical journals such as *The New England Journal of Medicine* and *Annals of Internal Medicine* take an important part in this analysis, too. As for the authors, Yasufuku K, with the H-index of 47 when we searched, had four studies published between 2004 and 2011 imported in our study, performing the best. These studies were mainly based on endobronchial ultrasound-guided transbronchial needle aspiration for lymph node staging of lung cancer, and compared different examinations such as positron emission tomography and computed tomography (CT) [14–17].

From the perspective of published design, we also study the topic distribution characteristics of the most cited articles. Two themes, BAL fluid along with Bronchoscopy and biopsy, occupy the primary part. Since the application of bronchoscopy in clinical practice, the two have been the focus of researches in human bronchial and lung diseases as an important means of bronchoscopy sampling and testing.

BAL is widely used to sample the lower respiratory tract from the perspective of clinical bronchoscopy, and materials obtained in this way are mainly alveolar contents such as respiratory mucous secretions [18]. BAL is a technique for sampling respiratory epithelial fluid, and analysis of the cellular and non-cellular components of the reflow may provide valuable information about airway inflammation [19]. BAL fluid collected through bronchoscopy can be used for bacteriological analysis to diagnose related diseases, such as ventilator-associated pneumonia [9, 20]. The keyword centrality analysis also shows that BAL had a high centrality in the 1990s and 2000s, showing such research direction is the absolute core, which is consistent with the research on subject words. These results suggest that BAL is still a bronchoscopy procedure that needs to be addressed in the future and that it is necessary to explore the drawbacks of this area, such as mucosal damage to the respiratory tract and invasive infections. Because of the invasive property, clinical workers should also be concerned about the possible damage to patients when using bronchoscopy for BAL fluid sampling in the future. Research and development of BAL sampling technology with more safety and comfort is also a possible direction in the prospective technology field.

In addition to BAL, bronchoscopy and biopsy are other keywords with high involvement. As an important method of respiratory tract sampling, bronchoscopy biopsy plays an important role in the diagnosis of lung cancer and other bronchial-related diseases, the fibreoptic bronchoscopy has been routinely used for the diagnosis of suspected lung cancer [21]. It is reported that bronchoscopy can be considered as the safest and most accurate tool for assessing the mucous membranes of the central and distal airways [22]. We also found in some studies that both bronchoscopies with or without a narrow range and radial endobronchial ultrasound (R-EBUS) were poorly diagnosed by the investigators for pulmonary lesions [23]. Thus, from a technical point of view, future work should focus on cutting edge technology to improve diagnostic accuracy.

As for the degree centrality analysis, there are differences between the 1990s and the 2000s. The hot words of the 1990s were those like BAL, inflammation, diagnosis, and biopsy. It is also consistent with the fact that there was a lot of attention paid to diagnosis in the 1990s. As for the 2000s, keywords with high-frequency became BAL, outcome, infection, and so on. Among them, it can be seen that researchers still pay high attention to the highest-frequency BAL. In addition, the focus of the 1990s on the diagnostic and technical aspects of treatment has shifted, with researchers focusing more on the evaluation of prognosis, infection control, and other aspects of treatment with bronchoscopy. Research on a number of treatments such as bronchial thermoplasty [24–26], bronchial valve implantation [27–29], and bronchoscopic removal of the tracheal foreign body [30] began to expand. Gradually, the study of bronchoscopy began to blossom.

The LCS represents the number of citations that a document has in the local data set. If a document has a high LCS value, we’d say it’s an important document in the special field and may even be a groundbreaking article [31]. Chronologically, we can notice these 30 articles obtained by using the LCS method in the 1000 most cited articles published from 1990 to 2020 actually ranged from 1999 to 2007, and even one-fifth of them presented in one single year, 2004. In connection with the previous analysis that the 2000s made the most articles in three decades, this interesting phenomenon may suggest that the research on bronchoscopy has been further expanded and deepened in these years. There is a consistency between the two methods because of 12 overlapping articles obtained. Throughout these 12 articles on the topic of research, more than half linked with E-BUS. This reflects a high research interest in this technology in the 2000s. For example, Yasufuku et al. pointed out in 2004 that lymph node sampling of hilum and mediastinum by EBUS-guided transbronchial needle aspiration (TBNA) technique was accurate and safe in diagnosing pulmonary diseases [14]. This research, in our analysis, has a citation number of 479 (Ranked 6th) and the LCS of 19. Electromagnetic navigation bronchoscopy (ENB) is another hot theme in 12 articles. It was concluded that ENB is a safe and effective diagnostic technique in the years around 2006 [32, 33], which has led to broad studies. These new techniques have undoubtedly received more and more attention, suggesting important directions for bronchoscopy research in the future.

We also took note of the progress of bronchoscopy-related research in recent years. Some researchers pointed out that bronchoscopic lung volume reduction using an endobronchial valve (EBV) is safe and effective in the treatment of chronic respiratory diseases such as emphysema. And with advances in one-way valve therapy, this is now a routine treatment option [34]; Bronchoscopic lung cryobiopsy, a novel biopsy method, was thought to have a meaningful effect on the multidisciplinary diagnosis of idiopathic pulmonary fibrosis [35]; Endoscopic ultrasound with bronchoscope fine needle aspiration (EUS-B-FNA) is a new technique which can be performed by interventional pulmonologists/thoracic surgeons with an echo-bronchoscope. With the help of EBUS, doctors can explore all the mediastinal lymph node stations in one endoscopic session, improving the accuracy in the diagnosis and staging of lung cancer [36]; Newer navigational modalities, such as robotic bronchoscopy and CT-guided cone beam bronchoscopy, might foreshadow the future in the bronchoscopic management of peripheral pulmonary lesions [37, 38]; Besides, a recent study illustrated the role of bronchoscopy in the management of hemoptysis, which still represents a frequent diagnostic challenge in routine clinical practice [39]. Those novel developments are so fantastic and instructive, while whether they are high-quality researches or not need more time to test. We will continue to monitor the progress of the discipline.

Admittedly, although our results produce some valuable information, which may play a certain role in suggesting the topic selection and research direction of intending researchers, there are also some limitations. First, as articles are filtered according to the number of citations, new publications that are significant in the field but have not yet reached high citation levels are ignored. Therefore, it tends to be a kind of retrospective study of historical articles. Second, this analysis included only published articles and excluded other papers like clinical guidelines, meeting notes, textbooks, meta-analyses, and reviews, which may lead to omission bias. Thirdly, although we did search without any language restrictions on Web of Science, some famous articles in languages other than English may have been omitted because of database limits. Last but not least, although we use bronchoscopy as the keyword to search for articles, we may miss some famous articles retrieved by other keywords.

## Conclusions

This article highlights the top 100 most cited articles in bronchoscopy over the past 30 years (1990–2020), including their time and geographical distribution, research topics, authors, research institutions, and research keywords. From the aspect of the research theme, we found that BAL and bronchial biopsies currently plays a major role. At the same time, they mostly focus on clinical trials, whereas basic laboratory research is inadequate, so more research on the subject is needed. In addition, because of the inherent limitation of our analysis, novel and instructive researches such as fluorescent bronchoscopy and advanced electromagnetic guided bronchoscopy may fail to enter our horizons. In summary, the field of bronchoscopy looks promising. With the advent of modern technology and easy access to different data, we can look forward to getting deeper researches in this area during the next 5–10 years.

## Supplementary information

**Additional file 1.**

## Data Availability

All data generated or analysed during this study are included in this published article and its supplementary information file.

## Abbreviations

BAL: Bronchoalveolar lavage
ANOVA: Analysis of variance
LCS: Local citation score
*AJRCCM*: *American journal of respiratory and critical care medicine*
CT: Computed tomography
R-EBUS: Radial endobronchial ultrasound
TBNA: Transbronchial needle aspiration
ENB: Electromagnetic navigation bronchoscopy
EBV: Endobronchial valve
EUS-B-FNA: Endoscopic ultrasound with bronchoscope fine needle aspiration
